# Draft Genome Sequence of the Vancomycin-Resistant Clinical Isolate *Staphylococcus aureus* VRS3b

**DOI:** 10.1128/genomeA.00452-17

**Published:** 2017-06-01

**Authors:** Suresh Panthee, Atmika Paudel, Hiroshi Hamamoto, Kazuhisa Sekimizu

**Affiliations:** a Teikyo University Institute of Medical Mycology, Hachioji, Tokyo, Japan; b Genome Pharmaceutical Institute Co., Ltd., Bunkyo-ku, Tokyo, Japan

## Abstract

We report here the draft genome sequence of the vancomycin-resistant strain *Staphylococcus aureus* VRS3b. The 2.8-Mb genome, assembled into 46 contigs, harbored 2,915 putative coding sequences. The G+C content of the genome was 32.7%.

## GENOME ANNOUNCEMENT

*Staphylococcus aureus* VRS3b was coisolated with *S. aureus* VRS3a from the exit site of a nephrostomy tube on a 64-year-old female in New York. As these two strains were isolated from the same patient, they were considered to be identical, and the characterization has been performed mainly for VRS3a. However, the vancomycin-resistant phenotype of VRS3b is more stable than VRS3a (1). Whereas the genome sequence of VRS3a (2) is publicly available, the VRS3b sequence has not been deposited in databases. In this study, we sequenced and analyzed the draft genome of strain VRS3a using the Ion PGM system.

*S. aureus* VRS3b was obtained from BEI Resources and grown at 37°C aerobically in tryptic soy broth containing 6 µg/mL vancomycin. Genomic DNA was isolated using a Qiagen DNA-blood minikit (Qiagen, Hilden, Germany) according to the manufacturer’s recommended protocol. One hundred nanograms of DNA were subjected to fragmentation using the Ion Xpress Plus fragment library kit (Thermo Fisher Scientific, Waltham, MA, USA) to prepare 400-bp reads according to the manufacturer’s recommended protocol. The libraries were then enriched in an Ion Chef (Thermo Fisher Scientific), and subsequent sequencing was performed using the Ion PGM system (Thermo Fisher Scientific). A total of 7 million reads were generated with a sequence coverage of 600-fold. The genome was first assembled using AssemblerSPAdes version 5.2.1.0 of the Ion Torrent Server (Thermo Fisher Scientific) and then analyzed with CLC Genomics Workbench version 9.5.3 (CLC bio, Aarhus, Denmark), resulting in 46 contigs greater than 1,000 bp. The combined length of the contigs was 2,819,384 bp, and the G+C content was 32.7%. The longest contig was 457,724 bp, an *N*_50_ was 166,923 bp and an *L*_50_ was reached in 5 contigs. The genome assembly was further analyzed using the Rapid Annotations using Subsystems Technology server (3), revealing *S. aureus* VRS1 as the most closely related sequenced organism. Annotation was performed using the NCBI Prokaryotic Genome Annotation Pipeline (4), which detected a total of 2,915 putative protein-coding sequences, 9 rRNAs, and 50 tRNAs.

Phenotypically, *S. aureus* VRS3b was reported to be resistant to multiple antimicrobial agents. This resistance was correlated with the presence of multiple genes responsible for resistance to antibiotics, such as methicillin (*mecA*), vancomycin (*vanA*, *vanZ*, *vanX*, *vanY*, *vanR*, *vanS*, and *vanH*), erythromycin (*ermB*), and beta-lactams (*blaZ*).

The draft genome sequence of *S. aureus* VRS3b can further be utilized to understand the mechanism and evolution of vancomycin-resistant *Staphylococci* spp.

### Accession number(s).

This whole-genome shotgun project has been deposited at DDBJ/ENA/GenBank under the accession number NBCP00000000. The version described in this paper is the first version, NBCP01000000.
